# Supplementary material for: Splice-Junction-Based Mapping of Alternative Isoforms in the Human Proteome
Source: Cell Rep. Author manuscript; Available in PMC 2020 Jan 15. (PMC6961840; doi:10.1016/j.celrep.2019.11.026)
Supplement: 2 [file NIHMS1546469-supplement-2.zip › DF1/PXD006675/MitralValve/MitralValve_12_ZC3H7B_AGVADQGTSNGLGSIDDIETGNVPDTR.pdf]

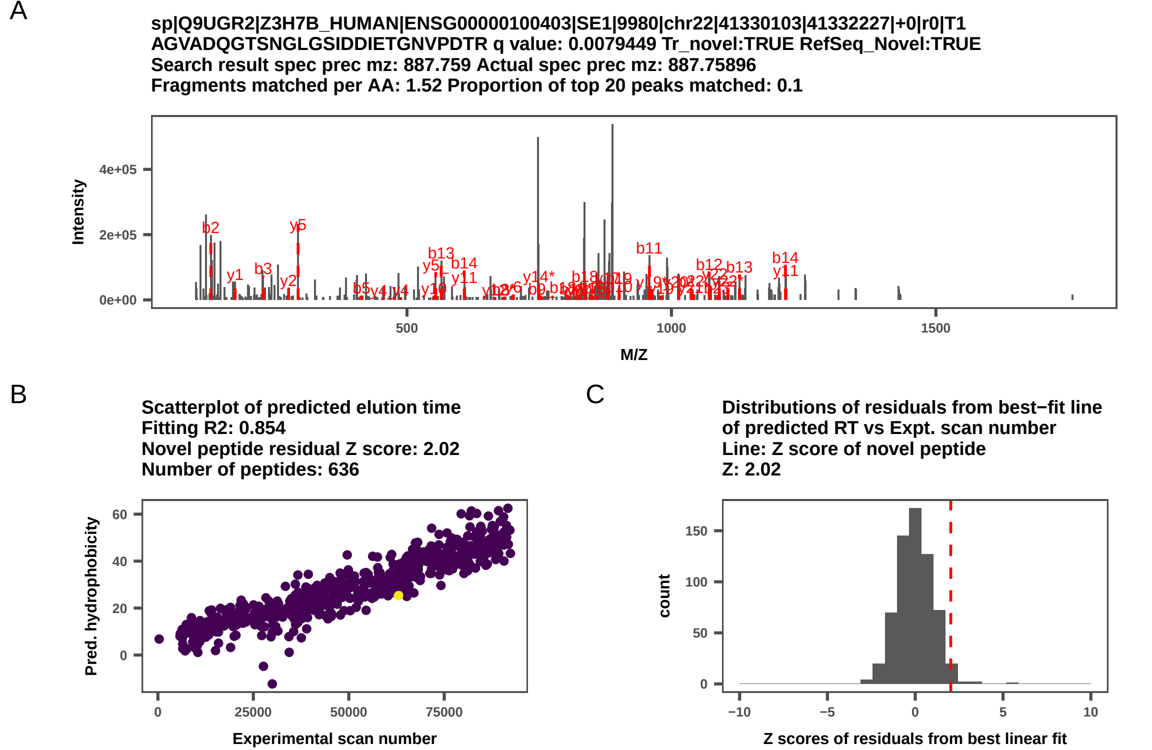
A
sp|Q9UGR2|Z3H7B_HUMAN|ENSG00000100403|SE1|9980|chr22|41330103|41332227|+0|r0|T1
AGVADQGTSNGLGSIDDIETGNVPDTR q value: 0.0079449 Tr_novel:TRUE RefSeq_Novel:TRUE
Search result spec prec mz: 887.759 Actual spec prec mz: 887.75896
Fragments matched per AA: 1.52 Proportion of top 20 peaks matched: 0.1
Intensity
4e+05
2e+05
0e+00
500
1000
1500
M/Z
B
Scatterplot of predicted elution time
Fitting R2: 0.854
Novel peptide residual Z score: 2.02
Number of peptides: 636
Pred. hydrophobicity
60
40
20
0
0
25000
50000
75000
Experimental scan number
C
Distributions of residuals from best−fit line
of predicted RT vs Expt. scan number
Line: Z score of novel peptide
Z: 2.02
count
150
100
50
0
−10
−5
0
5
10
Z scores of residuals from best linear fit
